# Übersehene Professionalität – individuelle Gelingensfaktoren von Peer- und Genesungsbegleitung

**DOI:** 10.1007/s00115-024-01754-x

**Published:** 2024-11-06

**Authors:** Daniela Schmidt, Imke Heuer, Madeleine Küsel, Guillermo Ruiz Perez, Sebastian von Peter

**Affiliations:** 1 Zentrum für seelische Gesundheit, dann Abteilung für Psychiatrie und Psychotherapie, Immanuel Klinik Rüdersdorf – Universitätsklinikum der Medizinischen Hochschule Brandenburg, 15562 Rüdersdorf, Deutschland Seebad 82/83,; 2 Abteilung für Psychiatrie und Psychotherapie, Universitätsklinikum Hamburg Eppendorf, 20246 Hamburg, Deutschland Martinistraße 52,

**Keywords:** Peer-Begleitung, Gelingensbedingungen, Erfolgsfaktoren, Persönlichkeit, Professionalität, Peer accompaniment, Conditions for success, Success factors, Personality, Professionalism

## Abstract

**Hintergrund:**

Auch durch die neue Personalverordnung wird die Peer- und Genesungsbegleitung zunehmend relevant. Dadurch stellt sich die Frage, welche individuellen Gelingensbedingungen es für die Implementierung von Peer- und Genesungsbegleitung gibt.

**Methode:**

Diese Arbeit ist ein Teilprojekt der vom Innovationsfonds finanzierten ImpPeer-Psy5-Studie, die bundesweite Anforderungen an die Implementierung von Peer- und Genesungsbegleitung in der krankenkassenfinanzierten Versorgung (SGB V) untersucht. 57 problemzentrierte Interviews mit Peer- und Genesungsbegleiter*innen (PGB), Mitarbeiter*innen (MA) und Nutzer*innen sowie eine darauf aufbauende Fokusgruppe wurden mithilfe einer vergleichenden thematischen Analyse untersucht. Diese Arbeit wurde vorrangig durch eine Forscherin umgesetzt, die über eigene Krisen- und Psychiatrieerfahrungen verfügt.

**Ergebnisse:**

Aus Sicht der befragten MA sind Sympathie und Persönlichkeit zentrale Faktoren für eine gelingende Implementierung der Peer- und Genesungsbegleitung. Demgegenüber stehen für die PGB ein professionalisierter Umgang mit der eigenen Krisen- und Recovery-Erfahrung sowie diverse Lebens- und berufliche Vorerfahrungen im Vordergrund.

**Schlussfolgerungen:**

Um die Impulse der Peer- und Genesungsbegleitung wirksam aufzunehmen, braucht es eine Anerkennung der professionellen Identität der PGB. Insbesondere Settings, die Aufgaben zwischen den Berufsgruppen weniger hierarchisch trennen und v. a. auf Beziehungsarbeit setzen, wie das der Zuhausebehandlung, sind für die Implementierung von PGB besonders geeignet.

## Abstract (en)

**Background:**

The peer support worker is becoming increasingly more relevant also due to the new personnel regulations. Therefore, the question arises which individual conditions for success are available for the implementation of peer support workers.

**Method:**

This study is a subproject of ImpPeer-Psy5 study financed by Innovation Funds, which investigates the nationwide requirements for the implementation of peer support workers in care financed by health insurance funds (German Social Act V, SGB V). Using a comparative thematic analysis, 57 problem-oriented interviews with peer support workers (PSW), employees and users as well as a focus group based on this were examined. This study was predominantly implemented by a researcher who already had experience in psychiatry and crises.

**Results:**

From the perspective of the employees interviewed, sympathy and personality are the core factors for a successful implementation of peer support workers. In contrast, a professional coping with one′s own crisis and recovery experience as well as diverse previous life and occupational experiences were prioritized by the PSWs.

**Conclusion:**

In order to effectively incorporate the impulse of peer support workers, a recognition of the professional identity of the PSWs is necessary. In particular, settings that separate the tasks between the professional groups in a less hierarchical manner and rely on building relationships, such as domestic treatment, are particularly suited for the implementation of PSWs.

## Hintergrund

Im Jahr 2016 wurde eine Richtlinie verabschiedet (PPP-RL), die Peer- und Genesungsbegleiter*innen (PGB) als neue Berufsgruppe explizit erwähnt, sodass die Frage ihrer systematischen Implementierung zunehmend relevant wird. In Deutschland bildet die EU-Förderung im Jahr 2005 die Grundlage für die Entwicklung von „Experienced-involvement“(EX-IN)-Qualifikationsmaßnahmen, in denen Personen mit eigenen psychischen Krisen- und Behandlungserfahrungen zu Genesungsbegleiter*innen ausgebildet werden. Außerdem gibt es Alternativen zur EX-IN-Ausbildung, wie z. B. Peer-Counseling-Kurse, bspw. die Interessenvertretung Selbstbestimmt Leben (ISL) und andere Programme, wie UPSIDES oder DBT Peer Coaching. Hierbei handelt es sich jedoch größtenteils zum einen um deutliche kleinere Initiativen, teils auch mit spezifischerer Zielgruppe (z. B: UPSIDES, DBT Peer Coach), zum anderen um Programme mit breiterem Fokus (etwa Peer Counseling ISL, das sich allgemein an Menschen mit Behinderungen richtet). Im Bereich der psychiatrischen Versorgung dominiert in Deutschland daher aktuell die EX-IN-Weiterbildung.

Im Zusammenhang stellt sich sowohl national als auch international die Frage, welche Gelingensbedingungen es für die Implementierung von Peer- und Genesungsbegleitung im klinisch-psychiatrischen Bereich gibt. Diese Frage ist vor allem deshalb relevant, weil die Peer-Begleitung im Ursprung außerhalb dieser Versorgungsbereiche und ursprünglich auch als nichtpathologisierender Gegenentwurf zu diesen entstanden ist [30]. Damit Peer-Begleitung erfolgreich und wirksam sein kann, ist es wichtig, dass die PGB ihre spezifischen Prinzipien und Vorgehensweisen selbstbestimmt anbringen und ausgestalten können [30]. Gerade diese Selbstbestimmung in den eigenen Vorgehensweisen von Peer- und Genesungsbegleitung ist jedoch nicht einfach zu untersuchen.

Diese Arbeit ist ein Teilprojekt der vom Innovationsfonds finanzierten ImpPeer-Psy5-Studie, die bundesweite Barrieren und Anforderungen an die Implementierung von Peer- und Genesungsbegleitung in der krankenkassenfinanzierten Versorgung (SGB V) untersucht [40]. Im Rahmen dieser Studie waren wiederholt die wechselseitigen Erwartungen von PGB und anderen Mitarbeiter*innen (MA) Thema sowie Fragen ihrer Zusammenarbeit. Im Einklang mit internationalen Untersuchungen [3, 6] stellten sich dabei Fragen wie: Woran liegt es, ob PGB in einer Versorgungseinrichtung ankommen oder nicht? Welche Faktoren spielen dabei eine Rolle, dass sie in den Teams toleriert werden? Sind eher professionelle Aspekte, wie sie bspw. in der Weiterbildung EX-IN vermittelt werden, oder eher Fragen des Mitmenschlichen entscheidend dafür, ob PGB „andocken“ können?

Anschließend an diese Fragen beschäftigte sich ein Teilprojekt der ImpPeer-Psy5-Studie mit den sog. „individuellen Gelingensfaktoren“ der Implementierung von Peer- und Genesungsbegleitung im krankenkassenfinanzierten Bereich. Dieser Begriff wurde aus internationalen Diskussionen zum Thema („individual success factor“) abgeleitet und überschreibt die Kompetenzen, Fähigkeiten, Eigenschaften und individuellen Merkmale, die für eine gelungene Implementierung von PGB oder anderen Interventionen hilfreich oder notwendig sind [13]. Zu diesem Thema gibt es national und international bisher nur wenig Literatur, weil die meisten Arbeiten zur Frage der Implementierung von Peer- und Genesungsbegleitung auf strukturell-organisationale Bedingungen fokussieren. Zumeist finden sich Aussagen darüber, dass die persönliche Erfahrung von MA mit einzelnen PGB Bedenken der MA gegenüber dem Konzept der PGB positiv und negativ beeinflussen können [3, 20, 21]. Andere Arbeiten betonen die Wertschätzung der Erfahrungsperspektive der PGB durch MA [1, 26 ]. Insgesamt bleibt die Frage, welche Fähigkeiten, Kompetenzen, Eigenschaften die PGB selbst mitbringen müssen, damit eine Implementierung in klinischen Kontexten gelingen kann, weitgehend unbeantwortet, sodass die vorliegende Arbeit sich diesem Thema widmet.

Zu diesem Zweck wurden in diesem Teilprojekt gezielt Interviews sowohl mit PGB als auch mit Mitarbeiter*innen, die mit ihnen zusammenarbeiten (MA) analysiert, um ihre Aussagen in Bezug auf individuelle Gelingensfaktoren miteinander vergleichen zu können. Hintergrund für diese Kontrastierung ist der Befund während der Auswertung des Materials, dass die Perspektiven darauf, welche Eigenschaften und Kompetenzen der Peer- und Genesungsbegleiter*innen zu einer gelungenen Implementierung führen, in den Sichtweisen der PGB von denen der MA deutlich abwichen. Außerdem wurde während der Auswertung deutlich, dass die Frage nach individuellen Gelingensbedingungen auch Antworten zu den kontextuellen Gelingensbedingungen einforderten, die, sofern relevant, aus diesem Grund ebenfalls aufgeführt werden. So wendet sich ein Ergebnisteil der (Nicht‑)Passung der beschriebenen individuellen Gelingensfaktoren mit den Versorgungsstrukturen zu.

## Material und Methoden

### Studiensetting und -design

Die Studie ImpPeer-Psy5 nutzte ein Mixed-methods(QUAL1-QUAN-QUAL2)-Design, um diverse methodische Ansätze und Perspektiven in den Austausch zu bringen. Neben einem systematischen Review, einer Reihe von Theory-of-change-Workshops und einer standardisierten Befragung (QUAN-Phase) – die durch Wissenschaftler*innen am Universitätsklinikum Hamburg-Eppendorf umgesetzt wurden – sind die beiden qualitativen Anteile (qualitatives Hauptprojekt [QUAL1] und diverse Teilprojekte zu vertiefenden Unterfragen [QUAL2]) an der Medizinischen Hochschule Brandenburg (MHB) umgesetzt worden. Eine Übersicht über die Gesamtstudie und mehr Details zu den unterschiedlichen Studienteilen findet sich bei Peter et al. [40].

Die Studie wurde mit dem Qualifikationsanbieter EX-IN Deutschland e. V. als Studienpartner durchgeführt. Außerdem wurde ein kollaborativer Forschungsansatz verwendet [37], was die Zusammenarbeit von Forscher*innen mit und ohne eigene Krisen- und Psychiatrieerfahrungen in allen Forschungsphasen bedeutet. Die Arbeit für das vorliegende Manuskript wurde vorrangig durch eine der Autor*innen umgesetzt (DS), die über eigene Krisen- und Psychiatrieerfahrungen verfügt. Sie analysierte den im Rahmen der QUAL1-Phase entstandenen Code-Baum in Bezug auf Fragestellungen zum Thema der individuellen Gelingensbedingungen und führte darauf aufbauend zusammen mit einer Teamkollegin eine Fokusgruppe durch.

Für die Impeer-Psy5-Studie wurde ein Ethikvotum an der Medizinischen Hochschule Brandenburg eingeholt (Nr. E‑01-20200826).

### Stichprobe

In beiden qualitativen Phasen wurden bundesweit Teilnehmende rekrutiert, in der QUAL-1-Phase (1) PGB, die in der krankenkassenfinanzierten Versorgung tätig sind oder waren, bspw. in psychiatrischen Krankenhäusern oder der Integrierten Versorgung, (2) Nutzer*innen (NU) von Peer- und Genesungsbegleitung und (3) MA diverser Berufsgruppen, die mit PGB zusammenarbeiten. Die Rekrutierung nutzte eine Kombination von Schneeballsampling und gezielter Fallauswahl, wobei ein ausgeglichenes Sample aus allen Bundesländern und Versorgungssettings anvisiert wurde (für mehr Details s. von Peter et al. [40]).

Für die vorliegende Arbeit wurde nur auf Material von MA und PGB zurückgegriffen, weil dieses, auch aufgrund der Anzahl der durchgeführten Interviews [s. Datenerhebung und -auswertung] am umfangreichsten (Tab. 1) war und außerdem die stärksten Kontraste im Verhältnis zur Forschungsfrage aufwies. Das Interviewmaterial von NU wurde zwar gesichtet, erhielt demgegenüber aber kein Material zur Frage der individuellen Gelingensbedingungen und wurde demzufolge nicht verwendet. Auch für die Rekrutierung der QUAL2-Phase für diese Arbeit erfolgte eine gezielte Fallauswahl aus der im Rahmen der QUAL1-Phase befragten MA und PGB-Gruppen. Dabei wurde auf eine größtmögliche Heterogenität im Hinblick auf berufliche und Erfahrungshintergründe, Geschlecht und Alter geachtet. Diejenigen MA und PGB, die während der QUAL1-Phase besonders ergiebiges Material zur Frage der individuellen Gelingensbedingungen geliefert hatten, wurden erneut angesprochen und zur Fokusgruppe eingeladen.

**Tab. 1:** Die Ergebnisse der Auszählung der relevanten Codes zum Thema individuelle Gelingensfaktoren aus QUAL1^a^

Kategorien	Definitionen	MA	PGB	NU
*Selbstbewusstsein*	*Selbstbewusstsein als Voraussetzung für die Tätigkeit Peer‑/Genesungsbegleitung*	*3*	*5*	*1*
Sympathie	Sympathie als Voraussetzung für die gute Zusammenarbeit/Akzeptanz im Team	8	0	1
Persönlichkeit	Empathie, „menschliche Qualitäten“, Individualität, Charaktereigenschaften	40	6	0
Berufliche Vorerfahrungen	Berufsausbildungen, Studien, Praktika, bisher ausgeübte Tätigkeiten	7	16	0
Professionalität	Zitate, die sich zur Professionalität der PGB äußern, professionalisierte Erfahrung, um in Kontakt zu kommen oder zu unterstützen in der Sicht der PGB	19	25	1
*Qualifikationen, Praktika etc.*	*Spezielle Qualifizierungsmaßnahmen zur Vorbereitung der Tätigkeit als PGB*	*5*	*6*	*0*
Belastbarkeit und Krisennähe	Geringe Belastbarkeit und Krisennähe als Barriere für ein gutes Gelingen	6	0	0

^a^ Die *kursiven* Zeilen markieren Kategorien, in denen sich die Anzahl der Aussagen ähnelt

### Datenerhebung und -auswertung

Zu Beginn der QUAL1-Phase wurden in einem mehrschrittigen Prozess sechs Interviewleitfäden entwickelt und in drei partizipativen Workshops abgestimmt (für mehr Details s. von Peter et al. [40]). Insgesamt wurden 57 problemzentrierte Interviews, mit 32 PGB, 19 MA und 6 NU durchgeführt, in Tandems, also durch jeweils zwei Forscher*innen mit und ohne Psychiatrieerfahrung. Die Interviews wurden aufgezeichnet und während der Transkription anonymisiert. Jedes Interview wurde von je zwei Teammitgliedern mit und ohne Psychiatrieerfahrung mithilfe der thematischen Analyse und MAXQDA induktiv kodiert. Die sich daraus ergebenden individuellen Codesysteme wurden in mehreren Schritten zu einem Code-Baum zusammengeführt, mit dem die Transkripte deduktiv recodiert wurden.

Während der QUAL2-Phase wurden aus dem QUAL1-Code-Baum gezielt Codes ausgewählt und für die Fokusgruppe während der QUAL2-Phase aufbereitet. Im Rahmen einer Voranalyse wurde deutlich, dass die Konstruktionen individueller Gelingensfaktoren der MA stark von denen der PGB abwichen. Aus diesem Grund wurden die Codes der befragten PGB getrennt von denen der MA analysiert, um Unterschiede und Gemeinsamkeiten beider Sichtweisen systematischer untersuchen zu können. Die Aussagen beider Gruppen der QUAL1-Erhebung wurden ausgezählt, um eine Gewichtung im Hinblick auf die zugeordneten Codes zu erhalten. Die Ergebnisse dieser Analyse flossen in den Leitfaden (s. Infobox 1) zur Umsetzung der Fokusgruppe (FG) ein. Der Austausch in den FG wurde aufgenommen, transkribiert und teils induktiv, teils deduktiv unter Zuhilfenahme der Codes aus der QUAL1-Phase ausgewertet.

#### Leitfaden Fokusgruppe

- Inwiefern spielt persönliche Sympathie für die Akzeptanz/Implementierung der Genesungsbegleiter*innen eine Rolle?
- Welche Rolle spielen die bisherigen Qualifikationen, Berufswege, Hobbys der Genesungsbegleiter*innen in der Ausübung der Tätigkeiten? Welche Rolle spielt die Qualifizierung durch EX-IN oder vergleichbare Träger?
- Welche individuellen Fähigkeiten halten Sie für wesentlich für die Tätigkeiten und das Ankommen im Team?
- Welche verallgemeinerbaren Skills brauchen Genesungsbegleiter*innen?
- Kann Genesungsbegleitung ohne die jeweilige Persönlichkeit gedacht werden, so wie in der Pflege, der Medizin?

Im Folgenden werden nur Themenkategorien besprochen, die sowohl von MA als auch PGB angesprochen wurden und für die inhaltliche oder graduelle Unterschiede zwischen diesen Gruppen auftraten. Das Ziel dieser Darstellungsweise ist eine ausführliche Behandlung jener Kategorien, in denen sich zentrale Unterschiede in der Gewichtung und im Bedeutungsinhalt zum Thema individuelle Gelingensfaktoren zeigten. Diese sind: „Sympathie“ und „Persönlichkeit“, „berufliche Vorerfahrungen“, „Professionalität“ und „Belastbarkeit und Krisennähe“ (Tab. 1). Wie oben schon angedeutet, ist in den Fokusgruppen der Blick zusätzlich auf Faktoren und Bedingung in den Versorgungsstrukturen geweitet worden. Auch, wenn sich hier die Perspektiven der MA und PGB nicht substanziell unterscheiden, sind diese Aussagen im letzten Ergebnisteil erhalten, weil sie sich spezifisch auf das Material der individuellen Gelingensbedingungen der QUAL1-Phase beziehen und diesbezüglich zusätzliche Aspekte einbringen.

## Ergebnisse

### Gelingensfaktoren aus der Sicht der MA und PGB

#### Sympathie und Persönlichkeit

Mitarbeiter*innen äußern in den Einzelinterviews (EI), dass die sympathische Ausstrahlung der PGB für das Gelingen der Integration ins Team positiv gewirkt habe:

„Ich glaube, es liegt daran, dass plötzlich im Team jemand sitzt, den ich total mag und der hatte eine Psychose und ich kenne sonst aber in meinem privaten Umfeld niemanden, der eine Psychose hat oder hatte oder auch schon mehrfach hatte. Ich kenne nur die Patienten. Und da ist in meinem Kopf die klare Trennung: Die da, ich hier. Und jetzt sitzt dieser Genesungsbegleiter neben mir, ich habe auch viel Spaß mit dem … nach dem Motto – ein ganz normaler Mensch … und ich nehme plötzlich diese Erfahrungsberichte von ihm ernster.“ (MA 54/EI)

Das gilt zudem teamübergreifend für die Wirkung von Peer- und Genesungsbegleitung im gesamten Setting:

„Die treffe ich dann im Aufzug, Kollegen, wenn sie sagen: ‚Ja bei mir in der Fortbildung, da war der Genesungsbegleiter. Das ist ja ein Netter!‘“ (MA 54/EI)

Demgegenüber ist die Thematisierung von Sympathie als Gelingensfaktor nur in den MA-Interviews zu finden und stellt kein Kriterium dar, das die PGB bedenken. Außerdem wird von MA der positive Effekt hervorgehoben, den die Persönlichkeit und das Einfühlungsvermögen der PGB auf die Arbeit mit den NU und die Zusammenarbeit hat:

„Die haben einfach ein sehr starkes Einfühlungsvermögen und haben eigentlich so gut wie immer auch aus dem Blickwinkel der Klienten argumentiert und gehandelt.“ (MA 70/EI)

„Ich glaube, das Entscheidende ist die Persönlichkeit. Und wenn was wichtig ist, dann... Dann sind das eher kommunikative, reflektive Techniken und Instrumente, die wesentlich entscheidend sind. Weniger theoretische Inhalte über Krankheitssymptome und solche Geschichten. Weil dafür gibt es ja andere. Da ist meine Berufsgruppe wahrscheinlich kompetenter, weil das ist unser Ausbildungsschwerpunkt.“ (MA 60/EI)

Diese Aspekte überwiegen dann auch in der Personalauswahl:

„Die Genesungsbegleiter, die ich so kennengelernt habe, da hatte ich den Eindruck, die waren durch ihre Persönlichkeit ganz gut dafür geeignet.“ (MA 65/EI)

Peer- und Genesungsbegleiter*innen messen ihre diesbezüglichen Qualitäten stets an der Fähigkeit, mit NU in Kontakt zu kommen und hilfreich zu sein:

„Die Fähigkeit, empathisch zu reagieren. In den Situationen, wo das nötig ist, mich auch komplett zurückzunehmen. Das fällt nämlich vielen schwer leider, stelle ich immer wieder fest.“ (PGB 26/EI)

„Was da vielleicht hilft, ist mein kreatives Denken.... Das hilft, meiner Meinung nach, jeden Tag bei der Arbeit, in den Gesprächen mit den Patienten. Und vielleicht auch achtsam durch die Welt gehen, dass mir viel auffällt in der Welt, was anderen vielleicht nicht so auffällt.“ (PGB 47/EI)

Die FG der QUAL2-Phase diskutierte die Fallstricke der Kategorien *Sympathie und Persönlichkeit* als Kriterium der Beurteilung:

„Es gibt total bescheuerte Sozialarbeiterinnen und es gibt auch total bescheuerte Genesungsbegleiterinnen. Das ist etwas, da wird manchmal bei der Genesungsbegleitung ganz anders drauf geguckt als bei anderen Berufsgruppen.“ (PGB D/FG)

„Wenn es einmal nicht klappt, dann würde man in einem Team niemals sagen, wir haben es mit einem Arzt probiert, machen wir nicht mehr, es gibt keine Ärzte mehr hier. Das würde niemand machen. Also was kann ich dafür, wenn es irgendwo einen Versuch gab mit einer Genesungsbegleiterin und es nicht funktioniert hat? Zu mir dann zu sagen, tut mir total leid, stellen wir nicht mehr ein, hat nicht funktioniert, das finde ich ganz schwierig. Wir sind keine homogene Gruppe, kennt man einen, kennt man alle. Wirklich nicht. Unser Kurs war so bunt. Es gab so viele verschiedene Menschen, aber auch so viele verschiedene Vorerfahrungen mit dem Berufsleben. Wir sind alles andere als homogen.“ (PGB D/FG)

#### Professionalität

Die *beruflichen Vorerfahrungen* der PGB werden von den MA eher weniger in den Vordergrund gestellt. Auffällig ist die Aussage eines MA, dass die beruflichen Qualifikationen im Vorfeld nicht nur nicht relevant, sondern auch hinderlich seien, wenn diese zu nah an klassischen Berufsbildern der Psychiatrie sind:

„Sie war Ergotherapeutin von Grundberuf und hat, ich glaube, damit gehadert, dass sie wenig Professionelles einbringen kann. Das war immer so ein Thema. Ich könnte doch auch das und das machen. Das wollten wir nicht. Also Ergotherapien-Vertretung oder so.“ (MA 57/EI)

Für die meisten PGB sind die *beruflichen Vorerfahrungen* demgegenüber essenziell. Sie werden stark kompetenz- und selbstbewusstseinssteigernd erlebt, vor allem aber aktiv in die tägliche Arbeit eingebracht. Seien dies Fähigkeiten zur Mediengestaltung, Erfahrungen als Hotelfachfrau oder ein absolviertes Fachstudium der Geschichte – alle werden von den PGB als hilfreich betrachtet, um mit NU ins Gespräch zu kommen oder ihnen ein hilfreiches Gegenüber zu sein:

„Ich habe Jugendlichen Nachhilfe gegeben ... Und da würde ich schon eher sagen, da erkenne ich Parallelen ... Bei den Patienten, die wirklich diese Zeit genutzt haben und länger zu mir gekommen sind, da entwickelt sich auch eine Beziehung. Und da ist es schon so, dass wir miteinander versuchen zu erarbeiten, wie das Leben möglichst lebenswert ist, obwohl es so ist wie es ist. Und das ist schon recht ähnlich wie mit der Nachhilfe. Da ist ja auch das notwendige Übel, dass man das alles nicht versteht und trotzdem irgendwie lernen muss.“ (PGB 17/EI)

Dies gilt auch für Zusatzqualifikationen:

„Ich habe vor der EX-IN-Ausbildung eine Sterbebegleiterausbildung gemacht. Und davon habe ich sehr viel mitnehmen können ... Weil es ganz viel mit Rücksichtnahme, mit sich zurücknehmen, dem Gegenüber den Raum schenken zu tun hat.“ (PGB 26/EI)

Dass MA diese persönliche Geschichte der PGB außerhalb der Krisenbewältigung seltener wahrnehmen, spiegelt sich auch in den Interviews der PGB.

„Also ich bin nicht ein Ungelernter, der Genesungsbegleiter wird. Aber ich glaube, das ist mein persönliches Ding. Ich habe da nicht irgendwie den Eindruck, man akzeptiert mich mehr, nur weil ich gelernter Werkzeugmechaniker bin oder mal eine eigene Firma hatte. Viele wissen das gar nicht, glaube ich.“ (PGB 03/EI)

Dennoch sprechen auch MA von *Professionalität *im Zusammenhang mit Peer- und Genesungsbegleitung. Dabei überwiegt der Respekt vor der Krisenerfahrungsperspektive, die als Dreh- und Angelpunkt der professionalisierten Erfahrung der PGB verstanden wird.

„Unvergleichbar, die eigene Erfahrung ... Ich kann schon bestimmte Dinge nachvollziehen, aber doch nicht in der Qualität, wie das Peerbegleiter können, und das ist das für mich häufig offensichtliche Alleinstellungsmerkmal ... das Alleinstellungsmerkmal ist, dass jemand diese Selbsterfahrung hat und durch die Qualifizierung auch noch mutmaßlich eine ganz gute Aufarbeitung dieser Selbsterfahrung erfahren hat.“ (MA 52/EI)

„Welche Rolle muss ich als Sozialarbeiter, als Arzt, als Psychologe erfüllen? Was wird auch von mir erwartet? Und dann zu merken, dass das banale Schmeißen eines Kissens [durch den PGB] einen super krassen Effekt hat, … es braucht nicht totale Eloquenz, es braucht nicht die super krasse Analyse, sondern die Begegnung in der Lebenswelt ist die Professionalisierung.“ (MA 82/EI)

Für PGB waren hinsichtlich der *Professionalität* nicht nur die Krisen-, sondern auch andere Erfahrungen relevant, um mit NU in Kontakt zu kommen oder zu unterstützen:

„Natürlich bin ich krisenerfahren. Aber das ist ein Teil von mir. Und ein anderer Teil hat eben zum einen, was weiß ich, vielleicht auch einen intellektuellen Hintergrund und einen Lebenserfahrungshintergrund. Und dann bin ich noch eine Mutter und so weiter und so fort, als dass ich das viel komplexer sehe, als jetzt nur zu sagen, Genesungsbegleitung, weil eine psychische Krise da war. Natürlich war die wichtig und prägend für mein Leben. Aber es macht einen ja mehr aus.“ (PGB 25/EI)

„Das ist eine große Lebenserfahrung von mir, dass ich mit wenig Geld supergut klarkomme. Es gibt ja auch diese Patienten, die waren super im Job. 3000 € verdient und dann in die Depression gekommen und Hartz IV nach drei Jahren, die gar nicht klarkommen mit so wenig Geld und sich das auch nicht mehr vorstellen, weil sie noch nie auf so einem niedrigen Standard gelebt haben. Und denen zeige ich dann, es geht. Wenn du da ein bisschen sparst, kannst du da wieder was reintun. Es ist nicht schön, aber du wirst nicht verhungern und du wirst auch deinen Stolz nicht verlieren. So geht es.“ (PGB 35/EI)

PGB betonen in diesem Zusammenhang den Wert des Wir-Wissens, etwas, das MA an ihnen eher nicht wahrnehmen:

„Na, ich bringe mittlerweile nicht nur meine eigene Krisenerfahrung mit ein, sondern ich bringe ganz viel an Kenntnis über unterschiedlichste Krisenerfahrungen ein. Das ist wieder der Begriff ‚Wir-Wissen‘, der ja nicht nur auf meinen Erfahrungen und meinen Emotionen und meinem Leben fußt, sondern den Horizont etwas erweitert.“ (PGB 37/EI)

#### Belastbarkeit und Krisennähe

Ausschließlich die befragten MA brachten die Themen Belastbarkeit und Krisennähe der PGB auf:

„Sie [eine PGB] war sehr dünnhäutig und oftmals selbst an ihren Krisen noch nah dran. Das muss überhaupt kein Problem sein. Wir haben auch gesagt, zieh dich zurück oder nutze das vielleicht sogar in Kontakt mit den Menschen hier und sag: Mensch, ich bin im Moment selbst dünnhäutig, vielleicht kann ich da auch auf ihre Krise ganz gut eingehen oder so.“ (MA 57/EI)

Zwar wurde das Einbringen von Krisenerfahrungen als wertvoll für die Arbeit mit den NU verstanden, aber diese Krisen durften nicht zu kurz zurückliegen bzw. wurde das Vorhandensein einer aktuellen Krise für die Arbeit in der Institution als kontraproduktiv verstanden:

„… aber natürlich [hat] auch seine persönliche Entwicklung da immer mit eine Rolle gespielt. Ob er stabil ist. Gerade die erste, die wir hatten, war einfach selbst noch nicht gesund. Das ist dann auch für die Patienten schwierig.“ (MA 72/EI)

Von einigen MA wurde angemerkt, dass eine potenziell fehlende Belastbarkeit der PGB nicht nur in Bezug auf die Arbeit mit den NU, sondern auch die Gestaltung der Arbeitsbedingungen berücksichtigt werden müsse:

„Dann tragen die beiden auch ein gewisses Päckchen mit sich rum, was sie auch noch bewältigen müssen, also privater Natur und sind auch im Grunde genommen nicht kerngesund. Da gibt es also durchaus immer wieder selbst Zeiten, die nicht so gut sind. Dem muss man auch Rücksicht entgegenbringen, sage ich mal so. Also man kann ja nicht sagen: ‚Nehmen Sie mal einen, der war mal psychisch krank, und den setzen Sie hier rein und damit ist der nicht mehr psychisch krank … das geht ja auch nicht und das ist ja klar, wenn man sich einen Mitarbeiterkreis aussucht, der beeinträchtigt ist.‘“ (MA 62/EI)

Aus dieser Annahme einer prinzipiellen Verletzlichkeit entstanden für einige MA auch Unsicherheiten, bspw. wie stark sie PGB bestimmte Tätigkeiten zumuten können oder nicht:

„Und ich weiß, dass es viele Kollegen gibt, die sich, bevor sie mit dem Genesungsbegleiter ins Gespräch gehen und auch während die mit dem im Gespräch sind, fragen: Kann ich ihm das jetzt zumuten oder wird er dann rückfällig?“ (MA 65/EI)

### Relevante Gelingensfaktoren im Setting

In der FG-Diskussion der Ergebnisse in der QUAL2-Phase wurde der Blick geweitet und es kamen vermehrt Aussagen über das Setting hinzu, in dem PGB angesiedelt sind. Es wurde deutlich gemacht, dass eine Betrachtung individueller Gelingensbedingungen allein nicht ausreicht, die Eignung von PGB zu gewährleisten. Einige Settings wurden als grundsätzlich geeigneter beschrieben, damit sich PGB entlang ihrer spezifischen Kompetenzen und Wissensformen einbringen können. Insbesondere die Eignung des Zuhausebehandlungssettings wurde hier intensiv diskutiert und der Behandlungsansatz des offenen Dialogs (OD). In beiden Fällen seien Aufgaben zwischen den Berufsgruppen weniger hierarchisch getrennt, bspw. im Vergleich zum stationären Setting und Professionalität zeige sich für alle Beteiligten stärker im Beziehungsaufbau:

„Aber ich finde, dass gerade die Begegnung in der Häuslichkeit einfach auch für alle Beteiligten, ob nun Fachpersonen oder Genesungsbegleiter, eine weit individuellere ist. Also man hat nicht so die Institution als Background, ob wohl oder übel, als Rahmen … Und da erscheint mir schon ein Unterschied zur Arbeit, zu dem klinischen Rahmen, wo jeder doch mehr in seinem Fachbereich bleibt … der Rahmen ist schon viel durchlässiger in dieser häuslichen Behandlung.“ (PGB B/FG)

„Bei uns ist mittlerweile eine Mehrheit der Kollegen im Open Dialogue geschult und zumindest diejenigen, die das tagtäglich Netzwerkgespräche machen, bei denen würde ich das genauso sagen. Das sind auch diejenigen interessanterweise, die am besten mit den Genesungsbegleitern bei uns klarkommen, also die auch am offensten dafür sind und bei denen es überhaupt kein Problem ist, mal eben schnell eine Praktikantin ins Team einzuschleusen.“ (MA C/FG)

Das Einbringen persönlicher Erfahrungen für das In-Kontakt-Treten werde außerdem vor allem besonders in Settings eingefordert, in denen im Sinne des offenen Dialogs gearbeitet werde:

„Und von daher erlebe ich das bei uns eher so, dass die Kolleg*innen, die im Open Dialogue geschult sind, die bringen das sehr bewusst in den Kontakt, sagen Dinge wie: ‚Ich bin auch Mutter‘, und so.“ (MA C/FG)

„Also ich würde das auch bestätigen, dass, denke ich, die systemische Grundhaltung auf jeden Fall förderlich ist, um alle Beteiligten mit ihren Erfahrungen zu integrieren.“ (PGB D/FG)

Gleichzeitig lasse eine Zusammenarbeit im offenen Dialog zu, dass sich PGB entlang ihrer spezifischen Kompetenzen und Erfahrungen einbringen könnten, auch weil dieser Ansatz vielfältige Zugänge und Herangehensweisen zulässt:

„Das heißt, obwohl ich vielleicht die Skills erwerbe im offenen Dialog, Fragen anders zu stellen, meine Haltung zu hinterfragen, anders auf das Gesagte einzugehen, habe ich natürlich trotzdem noch meine [PGB] Brille auf und bringe meine eigenen Inhalte ein. Und da würde ich dann schon sagen, es widerspricht sich nicht, solange die Haltung Einheit findet, sich als Individuum einzubringen.“ (MA 82/EI)

„Und wir haben auch immer irgendwie im offenen Dialog gearbeitet, sodass auch viele Klient*innen in die Gelegenheit kamen zu sehen, dass es tatsächlich unterschiedliche Ansichten gibt und dass sie sich selbst positionieren müssen, weil es kein Richtig oder Falsch gibt … Aber da sehe ich nicht so große Unterschiede zwischen Peers und nicht Peers.“ (MA 70/EI)

## Diskussion

Zusammenfassend lässt sich sagen, dass aus Sicht der befragten MA die Sympathie und Persönlichkeit zentrale Faktoren für eine gelingende Implementierung der Peer- und Genesungsbegleitung sind. Für den individuellen Erfolg von PGB in den Teams und darüber hinaus scheint ein emphatisches Einbringen der eigenen Fachlichkeit maßgeblich. Demgegenüber steht für die befragten PGB ein professionalisierter Umgang mit der Krisen- und Recovery-Erfahrung sowie diverse andere Lebens- und auch berufliche Vorerfahrungen sowie das Einbringen dieser Erfahrungen in die Kontaktaufnahme und -gestaltung mit den NU im Vordergrund, wenn es darum geht, individuelle Gelingensfaktoren zu priorisieren. Das Thema der fehlenden Belastbarkeit wurde dabei nur von den MA eingebracht, während dieses Thema in den Interviews mit den PGB nicht auftaucht. Die professionelle Identität der PGB kommt, wie diskutiert in der Fokusgruppe, vor allem in Settings zur Geltung, die mehr Beziehungsgestaltung und „Begegnung auf Augenhöhe“ durch alle Berufsgruppen einfordern bzw. zulassen, bspw. im Rahmen der Zuhausebehandlung.

### Professionelles Berufsbild?

Die Ergebnisse haben deutlich gemacht, dass die Erfahrungen mit dem Auftreten einzelner PGB das Bild der Peer- und Genesungsbegleitung prägen, wie dies auch in anderen Publikationen zum Thema beschrieben ist [3, 21]: Wenn einzelne PGB als „sympathisch“ oder „geeignet“ eingeschätzt werden, wird dieser Eindruck auf die ganze Berufsgruppe generalisiert. Im Hinblick auf die strukturellen Verhältnisse ist dies auch nicht verwunderlich, so sind die PGB die „Neuen“, sodass vorwiegend Einzelne, die in bestehende Zusammenhänge kommen, von den anderen MA im Hinblick auf ihre Passung oder Nichtpassung auf der persönlichen Ebene beurteilt werden [14]. Es geht aber auch darüber hinaus: Auch in der Literatur wird eine gute Beziehungsqualität zwischen PGB und dem Kollegium als enorm wichtig für die Implementierung der Peer- und Genesungsbegleitung betrachtet [1]. Damit werden Sympathie und die individuelle Persönlichkeit als Erfolgskriterium für die Akzeptanz oder das Scheitern des Modells der Peer- und Genesungsbegleitung in Anschlag gebracht. Dies ist problematisch, da diese keine berufsbezogenen und somit belastbaren Kriterien darstellen. Umgekehrt gibt es bisher in Deutschland kein festes Berufsbild, das als Instanz an sich wertgeschätzt und etabliert ist. Sympathie als Kriterium ist zwar zwischenmenschlich hilfreich, darf jedoch kein Kriterium für die alleinige Einschätzung der Kompetenzen und Eignung einer Berufsgruppe sein.

Neben menschlichen Kompetenzen liegen bei PGB auch fachliche Qualifikationen vor. In unseren Interviews wurden diese jedoch zumeist am Durchlaufen der Weiterbildung EX-IN oder vergleichbaren Angeboten festgemacht, Qualifikationskriterien, die auch in der Forschung als wichtig für die Implementierung erachtet werden [8, 22, 33]. Kommunikative und reflexive Techniken und Stärken werden sehr geschätzt. Der Fokus liegt in unseren Ergebnissen jedoch deutlich auf der Qualifizierung durch Krisenerfahrungen, was die PGB auf diese Erfahrungen festlegt, obwohl sie mit größerem Zeitabstand immer stärker in den Hintergrund treten können. Ein solcher Fokus lässt außerdem Qualifikationsmarken, die für andere Berufsgruppen ebenfalls eine Rolle spielen, in den Hintergrund treten, wie bspw. die berufliche Vorerfahrungen oder Möglichkeiten einer beruflichen Weiterentwicklung.

Schließlich bilden die Kriterien der Sympathie und Persönlichkeit viel Raum zum einen für eine Ungleichbehandlung im Verhältnis zu Mitgliedern anderer Berufsgruppen, zum anderen aber auch für Unsicherheiten in den Teams. Ungleichbehandlung ist hier in der Form des Rekurrierens auf die Persönlichkeit oder psychische Stabilität bei PGB zu verstehen, wenn es Schwierigkeiten gibt (oder auch wenn dies nicht der Fall ist [19]): Während bei den „Profis“ in diesen Fällen zumeist auf die Fachlichkeit Bezug genommen wird, scheinen bei den PGB die persönlichen Eigenschaften stärker in den Blick zu geraten. Auch dadurch wird die Professionalität von PGB zu wenig als solche verstanden, sondern die Passung einer PGB bleibt in der Wahrnehmung der MA bzw. der Institution ein individueller Glücksfall, während Negativerfahrungen immer auch auf das ganze Konzept Peer- und Genesungsbegleitung abzufärben drohen, wie dies im Ergebnisteil dokumentiert ist. Diesen Zusammenhängen ist dabei nicht zuträglich, dass die Qualifizierungsmaßnahme zur PGB deutlich kürzer als die Ausbildungszeiten der MA anderer Mitarbeitenden ausfallen und demnach oft nicht als gleichrangig anerkannt werden. Vielleicht hat gerade dieser Sachverhalt auch zu vielen der in der QUAL1-Phase erhobenen Einschätzungen der befragten MA geführt, was im Nachhinein empirisch nicht mehr überprüfbar ist.

In diesem Zusammenhang verwundert es nicht, dass das Thema Belastbarkeit nur von den MA aufgegriffen wird. Dieser Befund steht im Zusammenhang mit anderen Ergebnissen der QUAL1-Phase der ImpPeer-Psy5-Studie und der Literatur [20], die zeigen, dass PGB viel Stigmatisierung und Diskriminierung von den anderen MA erfahren. Die Zuschreibung einer fehlenden Belastbarkeit ist dabei ein häufig genanntes Phänomen und kontrastiert stark mit bspw. einer Arbeit aus dem deutschsprachigen Raum, die zeigt, dass PGB im Vergleich zu MA der anderen Berufsgruppen deutlich weniger häufig krankgeschrieben sind [17]. In unseren Interviews wurde darüber hinaus deutlich, dass Probleme in der Zusammenarbeit zwischen PGB und den anderen MA vor allem von den befragten MA oft an persönlichen Merkmalen, bspw. der fehlenden Belastbarkeit oder Sympathie, der PGB festgemacht wird, anstatt diese auf strukturelle Probleme zurückzuführen, bspw. Machtungleichgewichte in der Berufsgruppenhierarchie oder das Hinzutreten eines/r neuen Mitarbeiter*in in schon lange miteinander arbeitende Teams. Wenn immer Konflikte auftauchen, sollten diese also an erster Stelle auf strukturelle Hintergründe überprüft werden, anstatt sie vorschnell Eigenschaften oder individuellen Zuständen der PGB zuzuschreiben.

### Divergierende Konzepte von Professionalität und Erfahrung

Das Potenzial der Begegnung mit NU aus der Erfahrungsperspektive wird von sowohl den PGB als auch MA klar als Alleinstellungsmerkmal der PGB erkannt und findet auch in der Literatur Erwähnung [1]. Diese Fähigkeit, sich offen auf das Gegenüber einzulassen und alle Ebenen des eigenen Lebens zu nutzen, um in den Kontakt mit NU zu treten [15], wird von den MA jedoch nicht als Teil einer professionellen Identität, sondern als individuelle Besonderheit eingeschätzt. Dabei zeigen sich klare Unterschiede in der Definition und den Konzepten *Erfahrung* und *Professionalität* zwischen den Berufsgruppen: Was MA als individuelle Persönlichkeitsmerkmale formulieren, ist in den Augen der PGB ihre *professionalisierte Erfahrung aus allen Lebensbereichen*. Eine solche Sicht entspricht einem Recovery-Verständnis, wie dies von Amering und Schmolke beschrieben wird [2, 41]: Recovery ist ein ganzheitlicher Prozess, der alle Lebensbereiche umfasst und für PGB auch die Auseinandersetzung mit dem Krisenerleben anderer Menschen beinhaltet [11]. Ein Herauslösen oder die Konzentration nur auf Krisenerfahrungen greift hier zu kurz. Hier treffen Konzepte der fachlichen Spezialisierung und bedürfnisorientierter Begleitung aufeinander [30]. Die Reduktion der PGB auf überstandene und reflektierte Krisenphasen ihres Lebens verstellt den Blick auf den ganzheitlichen Ansatz von Recovery, den PGB verfolgen [38]. Dieses sog. „Wir-Wissen“ der PGB [23] steht eben nicht „nur“ für die eigenen Erfahrungen und Geschichte ein, sondern emergiert aus dem Engagement der PGB und ihrem Kontakt mit Mitstreiter*innen in der Selbsthilfe und/oder im Rahmen der Qualifizierung durch EX-IN oder andere Anbieter. Als Resultat entsteht gebündeltes Wissen, wie es im eigenen Leben und dem von anderen nicht geht und was wirklich sinnvoll sein kann [30, 35]).

In den Einzelinterviews und in der Fokusgruppe wurde sichtbar, dass PGB *alle* Lebens- und Berufserfahrungen für den Beziehungsaufbau mit den NU einbringen, dazu zählen Erlebnisse und Erfahrungen aus den Krisenzeiten ebenso wie Hobbys oder frühere Ausbildungen und Berufe. Während des ganzen Qualifizierungsprozesses der EX-IN-Kurse werden eigene Erfahrungen, Fähigkeiten und Möglichkeiten hinsichtlich der eigenen Stärken und Ziele schriftlich reflektiert, was unter dem Namen „Portfolioarbeit“ auch Bestandteil der Abschlussprüfung ist. Das Sehen und Wahrnehmen von eigenen Stärken, Erfahrungsschätzen und Reflexionsleistungen geht also Hand in Hand mit dem Einbringen derselben für die Begleitung von Menschen in psychischen Krisen. Das Portfolio mündet im persönlichen professionellen Profil: einer persönlich formulierten Ausarbeitung von „Meine Sichtweise, Mein Ansatz, Meine Fähigkeiten und Methoden“ (EX-IN-Kurrikulum) und stellt in diesem Zusammenhang ein gutes Instrument dar, auch um gegenüber anderen MA ein eigenes „professionelles Profil“ beschreiben zu können (https://www.trinetz.de/portfolio/).

In diesem Sinn unterscheidet sich die Portfolioarbeit auch von der professionell geleiteten Selbstreflexion, wie sie Ärzt*innen und Psychotherapeut*innen aus ihrer Ausbildung kennen. Während die Portfolioarbeit über das eigene Krisenerleben hinaus auch andere Fähig- und Fertigkeiten zum Thema macht, ist die Selbsterfahrung im Kontext der ärztlichen und psychotherapeutischen Weiterbildung eher auf eine deutlich fokussierte Biographiearbeit und die berufliche Reflexion gerichtet, oft auch mit dem Ziel, den Einsatz bestimmter therapeutischer Techniken und Ansätze in der Beziehung zu NU zu hinterfragen [4].

Verbunden mit den EX-IN-Aufbaumodulen, in denen es um die ganzheitlichen Aspekte der Begleitung geht, besteht das professionelle Qualifikationsprofil der PGB also aus einem vergleichbar umfassenden Netz aus Fachwissen, lebensweltlich reflektierten Erkenntnissen und einem Spektrum unterschiedlicher beruflicher und Lebenserfahrungen [24, 34]. Mit „professionellem Profil“ ist dabei gerade nicht die Ausrichtung auf oder Anpassung an das psychiatrische System gemeint, die von vor allem Peer- und User-Forschenden sorgenvoll als Vereinnahmung der Peer- und Genesungsbegleitung bezeichnet wird [12], sondern die Anerkennung des Besonderen dieses Wissens, dieser Erfahrungen und der daraus erwachsenden Kompetenzen der PGB als „professionell“.

Gleichzeitig haben PGB selbstverständlich eine individuelle Persönlichkeit, die MA auch wohlwollend wahrnehmen dürfen. Was noch zu wenig gesehen wird, ist die professionelle Identität, die zum einen im Wissen um Stärken und Erfahrungen, um die eigene Recovery-Erfahrung und zum anderen in der Fokussierung auf die Wirksamkeit der eigenen Recovery-Erfahrung für die NU besteht.

### Lösungsansätze

Lerbæk et al. machen darauf aufmerksam, dass psychosoziale Dienste durch einen starken Fokus auf Krankheit, Symptome und medizinische Behandlung gekennzeichnet sind, was schwierige Bedingungen für die Umsetzung einer Recovery-orientierten Praxis schafft [21]. Um den Impuls der Peer- und Genesungsbegleitung wirksam aufzunehmen, braucht es eine Änderung dieses Klimas [38] und insbesondere die Anerkennung der professionellen Identität der PGB. Dazu gehört zum einen eine stärkere Orientierung an neuen und wirksamen Ansätzen [5, 29] der Begleitung von Menschen in Krisen als dies für die Ausbildung der meisten Mitarbeiter*innen bisher gilt. Auch die Etablierung eines Drittels PGB in StäB-Teams, wie anderenorts gefordert [38], kann ein Weg sein, die Kultur einer Einrichtung zu verändern. Ein anderer Weg, der insbesondere für die stationäre Psychiatrie angezeigt ist, ist die Schulung von MA zur Reflexion und zum Einsetzen ihrer eigenen Erfahrungskompetenzen zugunsten der NU. Es ist gut, dass PGB für Empathie, Beziehungsarbeit und Erfahrungsexpertise geschätzt werden, es ist aber genauso wichtig, dass sie sich weiter professionalisieren und umgekehrt die „Profis“ auch Elemente der Peer-Begleitung in ihre Arbeit integrieren [26].

In der Fokusgruppe und auch in den Interviews wurde deutlich, dass vor allem Kolleg*innen, die im offenen Dialog, einer systemisch orientierten Begleitungsform mit hohen Recovery-Erfolgen ursprünglich aus Finnland [27], geschult sind, in ähnlicher Art und Weise wie PGB ihre persönlichen Erfahrungen im Kontakt mit den NU einbringen. Das liegt auch daran, weil der offene Dialog eine Form der Begegnung ist [39], die darauf zielt, Antworten auf psychotisches Erleben im Austausch unter allen an einer Krise Beteiligten zu finden und dadurch auf mehreren Ebenen einen bedürfnisorientierten Zugang zu NU ermöglicht [28, 39]. Der dem offenen Dialog eigene achtsame Umgang mit Sprache, dialogische und nutzerorientierte Prozesse der Sinnstiftung, die mit dem individuellen Netzwerk erarbeitet werden, haben auch Auswirkungen auf das professionelle Selbstverständnis und die Rolle von MA [28, 32]. Gefordert ist im offenen Dialog also eher der individuelle Mensch als das medizinische Know-how [39].

Diese Art der Weiterbildung ist eine Form der Professionalisierung, die allen MA der Psychiatrie offenstehen sollte, in den Interviews wurde dies auch als Desiderat in Vorbereitung oder zur Erleichterung der Implementierung von Peer- und Genesungsbegleitung bezeichnet. Sie erfordert eine Reflexion der eigenen Geschichte, Krisen und Lebensweisen in Weiterbildungen, ähnlich der Portfolioarbeit in EX-IN. Mehr noch, es braucht ein Klima der Offenheit, damit es möglich wird, mit „dem Eigenen“ sichtbar zu werden, denn es ist keine Ausnahme, dass Psychiatriemitarbeitende selbst therapeutische Hilfen in Anspruch nehmen, sich aber nicht trauen, diese Erfahrung im Kontakt mit den NU einzubringen [39]. Das Desiderat der Auseinandersetzung mit der eigenen Persönlichkeit und Geschichte wird von einem interviewten MA unserer Studie formuliert. Peer- und Genesungsbegleitung spiegelt den MA ihre Defizite in der Begegnung, sie wundern sich und bewundern, wie es den PGB gelingt, sich persönlich zu zeigen.

Ein zweiter und damit eng zusammenhängender Punkt, der ebenfalls im Rahmen der Fokusgruppe deutlich wurde: Der offene Dialog findet vor allem in der Zuhausebehandlung Einsatz. So nimmt es nicht wunder, dass für eine gute Integration von Peer- und Genesungsbegleitung und eine Anerkennung ihrer Professionalität insbesondere das nichtstationäre Setting angesprochen wurde, wie dieses auch andere Arbeiten tun [38]. Das Zuhausebehandlungssetting kann das Verständnis für Menschen in psychische Krisen verbessern und ein Gefühl der Zugehörigkeit und sozialen Integration vermitteln [7]. Im häuslichen Setting der NU ist jede/r individueller gefordert [38]. Es gibt weitaus weniger den Rückzug auf die eigene Fachlichkeit und weniger fachspezifische Aufteilung der Aufgabenprofile [38]. Neben den Vorteilen der Zuhausebehandlung für die NU zeigt sich hier die Chance, die unterschiedlichen Konzepte der Professionalisierung und Erfahrung von MA und PGB näher zusammenzubringen als es ein hierarchischeres Kliniksetting erlaubt.

Das bedeutet nicht, dass die Peer- und Genesungsbegleitung nicht auch im stationären oder teilstationären Setting wirksam sein kann. Dass dies der Fall ist, zeigen genug Belege [25, 42]. Außerdem machen Cleary et al. darauf aufmerksam, dass zur Anerkennung der spezifischen Professionalität der Peer- und Genesungsbegleitung auch ein neuer Ansatz des OD nötig ist, der die Stärken der PGB berücksichtigt und ihre Ansätze mit bisherigem OD-Konzepten verzahnt [9]. Vielleicht weist der Peer Supported Open Dialogue (POD), so wie er derzeit im Rahmen der vom NHS breit finanzierten ODESSSI-Studie umgesetzt und evaluiert wird, hier neue Wege [16, 31].

### Schlussbemerkungen

Es wurde deutlich, dass für PGB und MA jeweils eigene individuelle Gelingensfaktoren im Vordergrund stehen. Als mögliche Lösung wurde diskutiert, dass sich Zuhausebehandlungsformen, und insbesondere auch der offene Dialog, dazu eignen können, um mit diesen Unterschieden konstruktiv umzugehen. Peer- und Genesungsbegleitung ist durch die Arbeitsweise geprägt, Menschen in verstörenden Krisen auf der Grundlage und durch eine Vielfalt an beruflichen, lebensweltlichen und auch Krisenerfahrungen zu begleiten. Sie wirkt deshalb als Vorreiter und ist besonders dort wirksam, wo PGB auf ein Umfeld treffen, das diese Art der Begleitung ebenfalls bejaht und/oder sich zu eigen macht. Eine eigene psychiatrische Krisengeschichte ist etwas, das MA der Psychiatrie zumeist nicht selbst erlebt haben oder offengelegen wollen. Sich jedoch als Persönlichkeit sichtbar und nahbar zu machen, steht ihnen ebenso offen.

## Data Availability

Die erhobenen Datensätze können auf begründete Anfrage in anonymisierter Form beim korrespondierenden Autor angefordert werden. Die Daten befinden sich auf einem Datenspeicher an der Medizinischen Hochschule Brandenburg.
